# Effect of Nb Content on Cyclic Oxidation Behavior of As-Cast Ti-1100 Alloys

**DOI:** 10.3390/ma13051082

**Published:** 2020-02-29

**Authors:** Yingjun Song, Binguo Fu, Tianshun Dong, Guolu Li, Fei Wang, Xuebo Zhao, Jinhai Liu

**Affiliations:** School of Materials Science and Engineering, Hebei University of Technology, Tianjin 300130, China; syj15522011070@163.com (Y.S.); dongtianshun111@163.com (T.D.); liguolu@hebut.edu.cn (G.L.); 18822028669@163.com (F.W.); zhaoxueboliu@126.com (X.Z.)

**Keywords:** Ti-1100 alloy, Nb content, oxidation behavior, surface morphology

## Abstract

The cyclic oxidation behaviors of the as-cast Ti-1100-xNb (x = 0.5, 1.0, 1.5, 2.0) alloys exposed at 650 °C for up to 100 h were systematically investigated. The aim of this work is to explore the in-depth oxidation mechanism by using the oxidation kinetics and the structure of the oxide products. The oxidation kinetics were determined by thermogravimetrically, and the microstructure and composition of the oxidation scale were studied by using XRD and SEM. The results demonstrate that Nb can significantly improve the oxidation resistance. However, the average weight gains of the alloys decrease firstly and then increase with the increase of Nb content. The oxidation kinetics obeys a parabolic model. The Ti-1100-1.0Nb alloy has the lowest k_p_ value, which is 5.7 × 10^−13^ g^2^cm^−4^s^−1^. The surface oxidation products are mainly composed of massive or acicular rutile-TiO_2_, Ti_x_O (x = 3, 6), NbO_2_ and Al_2_O_3_. Besides, Al_2_(MoO_4_)_3_ oxide is also presented on the oxidation surface of the Ti-1100-1.5Nb alloys. Ti-1100-1.0Nb alloy shows the best oxidation resistance property revealed by combining weight gains and EDS-SEM element content profiles analysis. The interaction of Nb, O, Ti, and other elements retarded the diffusion of O atoms into the alloys, which improves the oxidation resistance.

## 1. Introduction

Ti-1100 alloy (Ti-6Al-2.7Sn-4Zr-0.4Mo-0.45Si) as a near α titanium alloy, has certain potential to be used in high temperature parts of aero-engine because of its excellent high temperature strength and creep resistance and corrosion resistance up to 600 °C [1,2]. Previous papers have reported that the room temperature strength of the forged Ti-1100 alloys can reach 900–950 MPa, and the high temperature (593 °C) strength can reach 500–570 MPa [3,4]. With the development of aircraft toward high flight speeds, in addition to low density and high strength, titanium alloys are also required to withstand higher temperatures. While a limitation of developing near-α titanium alloy is that the high temperature oxidation resistance is significantly reduced when the temperature exceeds 600 °C, the severe surface oxidation of high-temperature components of aeroengine will result in the decrease of effective load bearing area, which causes structure failure [5,6]. Previous investigation has reported that adding Nb to titanium alloy could improve the high temperature oxidation resistance and mechanical properties [7]. So, the addition of Nb element to Ti-1100 alloy may be a method to improve its high temperature oxidation resistance.

The oxidation process of titanium alloy is mainly affected by time and temperature [8], including two parts: one is the internal diffusion of O to the matrix, which can promote the formation of the low valence titanium oxide (Ti_x_O, x = 6, 3, 2) [9]. The other is the formation of TiO_2_ oxide particles on the oxidation surface. When the oxidation temperature exceeds 500 °C, the porosity of oxidation surface increases, even the TiO_2_ particles dissolve [10]. Researches showed that the addition of 5–10 at.% Nb could improve the oxidation resistance of Ti-Al alloys [11,12]. The effects of Nb atoms on oxidation behavior could be summarized as [13,14,15,16]: (1) Nb replaced the Ti^4+^ in TiO_2_, leading to a reduce of O^2−^ vacancy, which hampered the diffusion of O ion. (2) Nb could improve the activity of Al and promote the formation of the density Al_2_O_3_ oxide film in TiO_2_ oxide-layer, which can reduce the solubility of O atoms in oxide-layer. (3) Nb could reduce the solubility of O in α-Ti phase lattice. However, the solid solubility of Nb in titanium alloys is limited, and the interaction of Nb with Al, Sn, Zr, Mo, and Si in the Ti-1100 alloy can form the secondary phase and change the surface oxide structure [7]. With increasing Nb element, excessive Nb segregates in the grain boundary, which may result in the oxide scales cracking and high temperature oxidation resistance depreciation [8,17]. The content of Nb in the near α titanium alloy is usually between 0.7–1.0 wt.% [18], and the excess Nb content can be selected as 1.5 and 2.0 wt.%.

Thus, the effect of Nb content (0.5, 1.0, 1.5, 2.0 wt.%) on cyclic oxidation behavior of as-cast Ti-1100 alloys was systematically investigated in this present work. The aim of this study is to explore the in-depth oxidation mechanism from the oxidation kinetics, surface oxidation products, and the cross-section oxidation layer elements distribution.

## 2. Materials and Methods 

The Ti-1100-xNb (x = 0.5, 1.0, 1.5, 2.0) alloys were prepared by a vacuum arc smelting furnace with a non-consumable tungsten electrode and water-cooled copper crucibles. The melting stocks weighing 40 g were melted in a sealed chamber, and the melting process was at a controlled atmosphere of dry high purity argon (99.99%) maintained at 10,000 Pa. Each specimen was smelted four times to make the composition uniform. The chemical compositions tested by fluorescent spectrometer are given in Table 1.

The oxidation specimen with a dimension of 10 × 10 × 3 mm was cut by wire-cut electrical discharge machining (WEDM), which used electrical impulses to shape and manipulate hard metal materials. WEDM is mainly composed of machine itself, numerical control (NC) system, impulse power, working liquid cycle system, and machine accessories and so on. All surface roughness of the specimen is not less than 0.5 μm after grinding and polishing process. Before performing the oxidation test, the specimens were ultrasonically cleaned using the acetone solution to strip away the oily and oxidation particles. The following steps needed to be considered: (1) The size of the test specimen was measured by the micrometer with an accuracy of 0.01 mm to calculate the surface area; (2) Each specimen needed to be put in a cylindrical corundum crucible and dehydrated at 100 °C for 30 min; (3) The weight of the crucible and specimen was weighted by the CP224C electronic balance (Ohaus, USA) with an accuracy of 0.01 mg as the initial weight before oxidation test.

Cyclic oxidation experiment was performed in a resistance furnace (KSL-1100X type) (HF-Kejing, Hefei, China) at 650 °C for up to 100 h. The crucibles were taken out every 12 h to measure the weight gains, and then put it back into the furnace to continue the cyclic oxidation test. In order to keep reproducibility, three specimens in each composition alloy were tested, and the statistically averaged values of the weight gains were obtained to analysis the oxidation kinetics. The Nikon Eclipse MA100 type optical microscope (OM) (Nikon, Kyoto, Japan) was used to observe the microstructure of as-cast alloys, and the Quanta 450-FEG type scanning electron microscope (SEM) (FEI, Eindhoven, Netherland) with an octane plus type energy dispersion spectrometer (EDS) was used to examine the oxidation surface and cross morphology. The phases and oxidation products of the as-cast alloys were examined by the D/Max-2500/pc type X-ray diffractometer (XRD) (Rigaku, Akishima, Japan) with copper Kα radiation, and the 2θ scans were collected from 10° to 90° with the scanning speed of 8°/min. The obtained data were analyzed by MDI Jade 6 software. Specimens for OM observations were prepared according to conventional metallographic techniques. The specimens were polished and etched with Kroll’s reagent (6 vol.% HNO_3_, 3 vol.% HF, 91 vol.% H_2_O).

## 3. Results and Discussion

### 3.1. Microstructure

**Figure 1:**
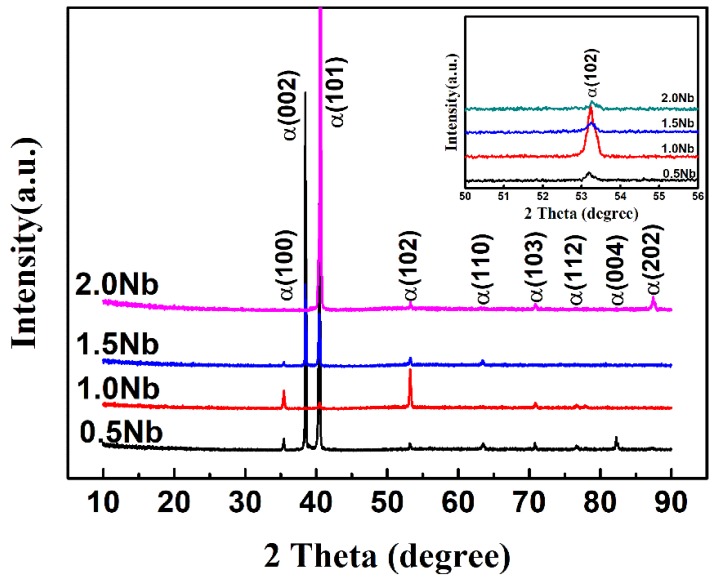
XRD patterns of the as-cast Ti-1100-xNb alloys.

XRD spectra of the as-cast Ti-1100-xNb (x = 0.5, 1.0, 1.5, 2.0) alloys are shown in Figure 1. It can be observed that all the peaks are well matched with the α-Ti phase regardless of Nb content. Nb is a β-Ti stabilizing element, however no β phase is detected due to the sensitivity of XRD, and the alloy is still classified as near α titanium alloy. It also can be seen that the XRD peaks move to a low angle direction and then shift toward high angle orientations with the increase of Nb content. According to Bragg’s equation, the shift in peaks will cause an increase or decrease in the α-Ti phase lattice parameter. The effect of Nb content on the α-Ti lattice constant of the as-cast Ti-1100-xNb alloys will be discussed in the later part.

**Figure 2:**
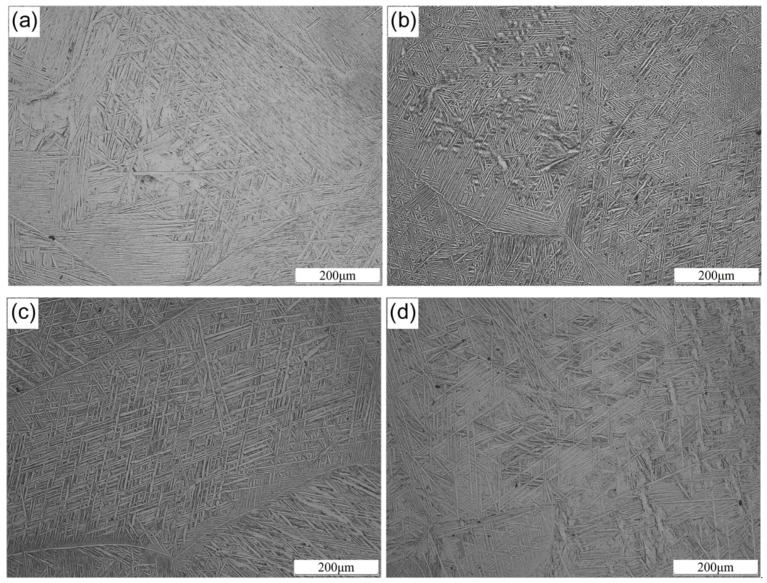
OM microstructures of the as-cast Ti-1100 based alloys (**a**) Ti-1100-0.5Nb; (**b**) Ti-1100-1.0Nb; (**c**) Ti-1100-1.5Nb; (**d**) Ti-1100-2.0Nb.

Figure 2 shows the OM microstructure of the as-cast Ti-1100 based alloys. It can be seen that addition of Nb did not change the microstructure classification of the alloys. All the microstructures are the widmanstätten structure with typical basket weave features. The average α-laths spacing of the alloys is 2.3, 1.4, 1.7 and 1.9 μm, respectively, which calculated by a mean linear intercept method. With the increase of Nb content, the space between α-laths decreases first and then increases. Our previous investigation found that the addition of 0.5 wt.% and 1.0 wt.% Nb had a grain refinement effect on Ti-1100 alloys [7]. However, the excessive Nb would weaken the grain refinement effect. This is due to the fact that Nb is a β-Ti stabilizing element, and its solid solubility in the β-Ti phase is much greater than that in the α-Ti phase [19]. With the further increase of Nb content, the amount of β-Ti phase increase, and the solid solubility of Nb in the β-Ti phase increases, leading to the weakening of the pinning grain boundary effect. Therefore, the α-laths spacing began to coarsen.

### 3.2. Analysis of Oxidation Kinetics

**Figure 3:**
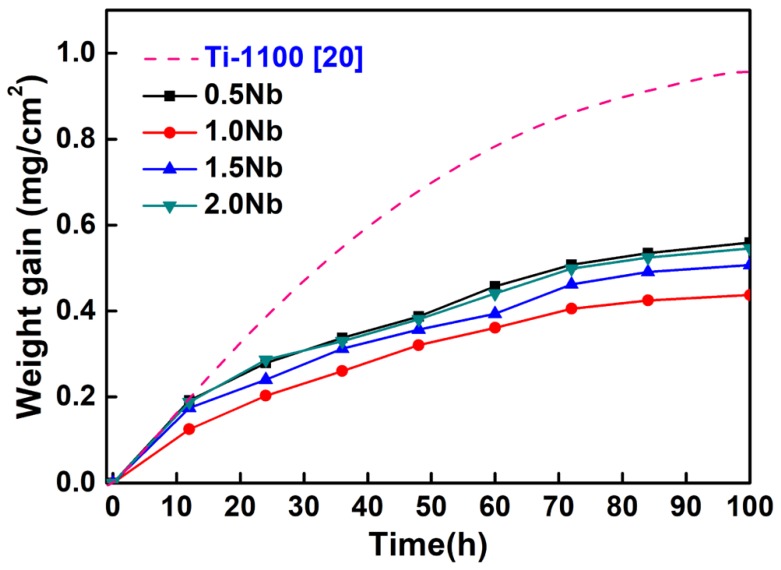
Variation of average weight gain with respect to oxidation time of the investigated alloys oxidized at 650 °C.

Figure 3 refers to the average weight gains per unit oxidation surface (ΔW/A) of Ti-1100-xNb (x = 0.5, 1.0, 1.5, 2.0) alloys and Nb-free Ti-1100 alloy [20] cyclic oxidized at 650 °C in an atmospheric environment for up to 100 h. The results indicate that all the average weight gains of the same composition alloy increase with the increase of oxidation time. The average weight gains of the alloys for 100 h oxidation decrease firstly and then increase with the increase of Nb content, and its value is 0.89, 0.56, 0.43, 0.51, and 0.55 mg/cm^2^, respectively. All the weight gains of Nb-added alloys are lower than that of Nb-free alloys, which shows that Nb can significantly improve the oxidation resistance. It also can be seen that the weight gain curves of the alloys follow a parabolic oxidation kinetic model, indicating that the oxidation process was controlled by diffusion [21]. The oxidation kinetics can be described by the Equation (1) [21,22]:

(1) (ΔW/A)^n^ = k_n_t

where ΔW/A is the weight gain per unit surface area, n is the oxidation reaction index, k_n_ is the oxidation rate constant, and t is the time.

According to the regression analysis of the curves in Figure 3, the values of the oxidation reaction index (n) were presented in Table 2. When n = 1, the oxidation kinetics curve followed the liner law, and if n = 2, the oxidation kinetics curve followed the parabolic law [21]. It can be seen that all the values of n are close to 2, and the corresponding coefficient of determination R^2^ of n values are 0.996, 0.989, 0.995 and 0.995, respectively. Therefore, the parabolic model was confirmed to fit well with the oxidation kinetic behavior.

**Figure 4:**
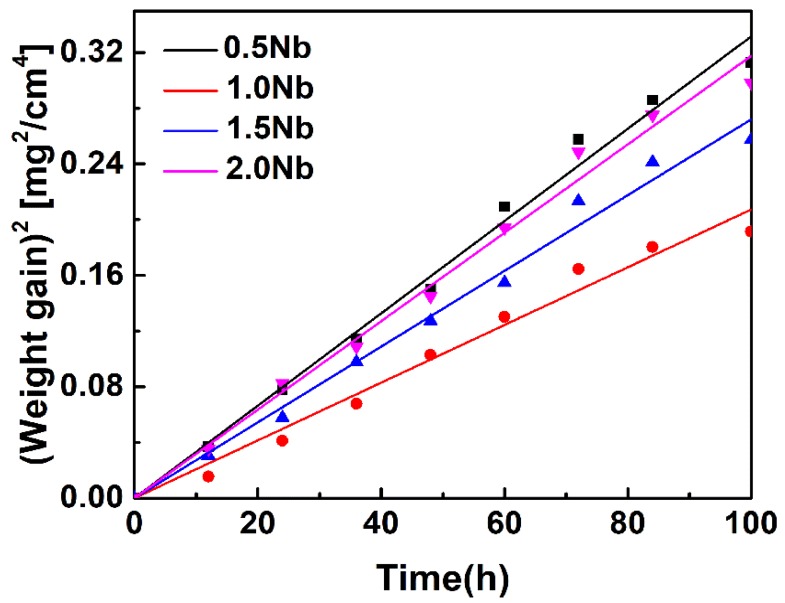
A plot of (ΔW/A)^2^ vs. oxidation time for obtaining the parabolic oxidation rate constant.

Figure 4 shows a plot of (ΔW/A)^2^ versus oxidation time for determination the parabolic oxidation rate constant (k_p_) of the investigated alloys. k_p_ values can be obtained from the slope of regression curves of Figure 4 and the results are presented in Table 3. It can be seen that k_p_ values decrease firstly and then increase with the increase of Nb content. The corresponding coefficient of determination R^2^ of k_p_ values are 0.990, 0.981, 0.988 and 0.989, respectively. The Ti-1100-1.0Nb alloy has the lowest k_p_ value, which revealed the best oxidation resistance property.

### 3.3. Phase Composition of the Oxidized Surface

**Figure 5:**
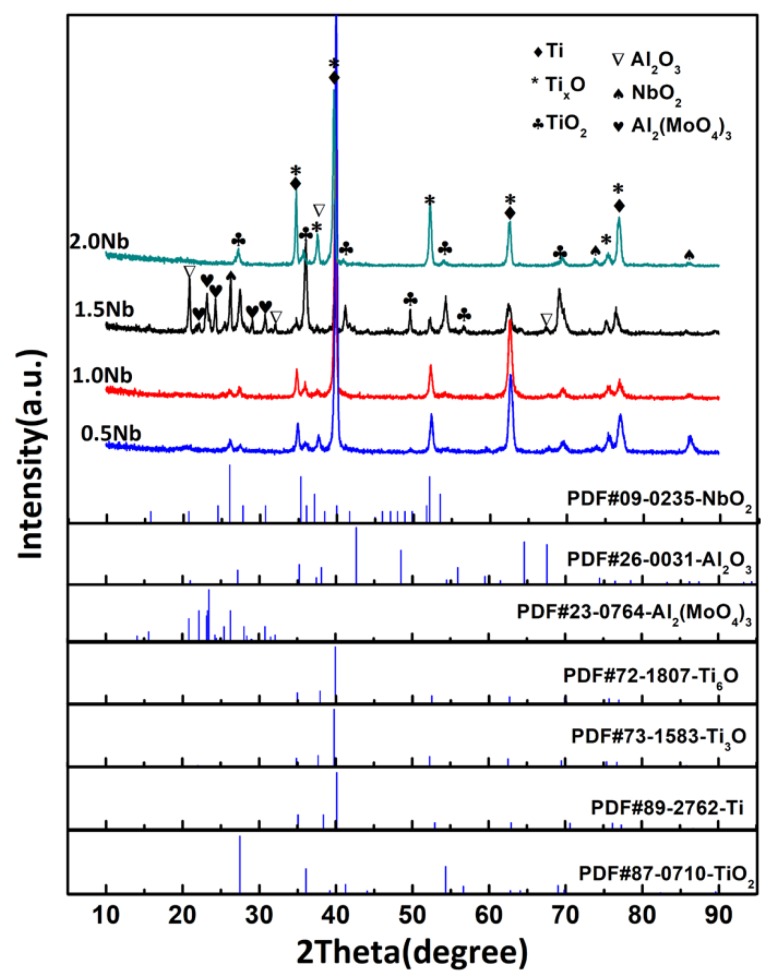
XRD patterns of the Nb contained Ti-1100 alloys surface after oxidation during 100 h at the temperature of 650 °C.

**Figure 6:**
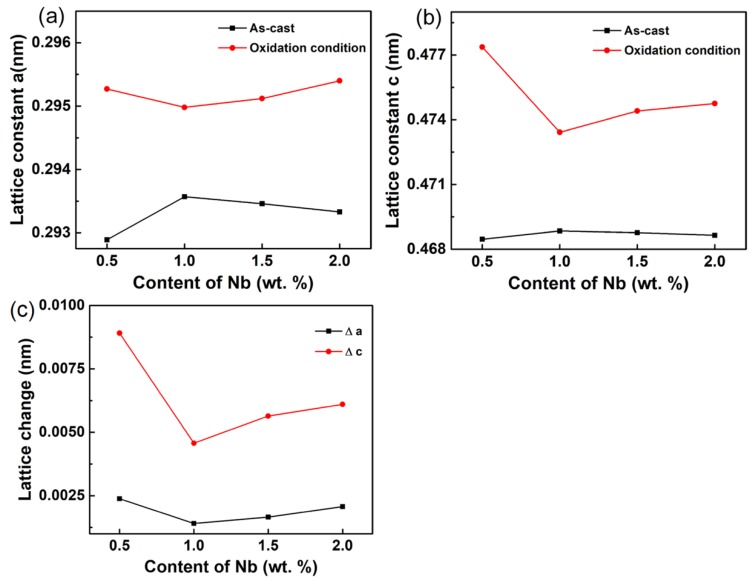
Effect of Nb content on the α-Ti lattice constant (**a**) a; (**b**) c and (**c**) lattice change of the as-cast Ti-1100-xNb alloys before and after oxidized at 650 °C for 100 h.

Figure 5 shows the XRD patterns of oxidized surface of the Ti-1100-xNb (x = 0.5, 1.0, 1.5, 2.0) near α titanium alloys under oxidation at 650 °C for 100 h. Rutile-TiO_2_, Ti_x_O (x = 3, 6), NbO_2_, Al_2_O_3_, and Al_2_(MoO_4_)_3_ were detected as major oxidation products on the surface. It also can be seen that the peak intensity in Figure 5 changes irregularly, and the local unusual peak intensity may be caused by preferred crystallographic orientation, or residual stress during the oxidation process, which needs further study. The lattice constant of α-Ti of the as-cast Ti-1100-xNb alloys before and after oxidized at 650 °C for 100 h are calculated by XRD refinement, and the results are presented in Table 4 and Figure 6. It can be seen that both the lattice parameters ‘a’ and ‘c’ of the as-cast alloys first rise and then decrease, with the increase of Nb content. Nb can replace titanium atoms in α-Ti lattice to form substitutional solid solution inducing an increase in the lattice parameter. However, the continue addition of Nb decreases the silicon solid solubility in Ti to promote the formation of β and silicide phases, which caused the α-Ti lattice contraction [7]. When the as-cast Ti-1100-xNb alloys are oxidized at 650 °C for 100 h, both the lattice parameters ‘a’ and ‘c’ increase due to the small size interstitial oxygen dissolving into the lattice. And the lattice expansion in the c-axis direction is more severe (Figure 6c), which is consistent with the findings of Baillieux et al. [23]. Jostsons et al. showed that the solution of O atoms in α-Ti lattice can reach 34 at.% [24]. It is also found that the Ti-1100-1.0Nb has the minimal lattice changes (Figure 6c), which shows the best lattice stability.

The massive solid solutions of O atoms in α-Ti can promote the formation of the low valence titanium oxide Ti_x_O (x = 3, 6) near the matrix surface, and the XRD analysis has proved the existence of Ti_3_O and Ti_6_O (Figure 5). Thermodynamic Equations (2)–(4) [25] at 650 °C showed that NbO_2_ was easy to form but with the lower stability, however the Al_2_O_3_ was more difficult to be formed in spite of the best stability. The lower Nb content and oxidation temperature caused the weak peak intensity of NbO_2_ and Al_2_O_3_.

(2) Nb (s) + O_2_ (g) = NbO_2_ (s) ΔG = −626.1 kJ/mol

(3) Ti (s) + O_2_ (g) = TiO_2_ (s) ΔG = −776.0 kJ/mol

(4) 2 Al (s) +3/2 O_2_ (g) = Al_2_O_3_ (s) ΔG = −1386.3 kJ/mol

With the increase of Nb content, the diffraction peak intensity of TiO_2_ and Al_2_O_3_ became stronger, and the peak of Ti_x_O constantly decreased. This phenomenon illustrated that the solution interstices of oxygen are captured by Nb atom, and the research shows that Nb atoms may take place the Ti atoms in the α-Ti lattice and it can promote the formation of TiO_2_ and Al_2_O_3_ [10]. The Al_2_(MoO_4_)_3_ phase was first discovered in oxidation surface of Ti-1100-1.5Nb oxidation at 650 °C, and its peak is stronger. The standard Gibbs free energy of formation Al_2_(MoO_4_)_3_ at 650 °C (Equation (6)) can be obtained from the thermodynamic Equations (4) and (5) [25]. The negative value proves that the Al_2_(MoO_4_)_3_ phase can be formed spontaneously.

(5) Mo (s) + 3/2 O_2_ (g) = MoO_3_ (s) ΔG = −626.1 kJ/mol

(6) 2 Al (s) + 3 Mo (s)+ 6 O_2_ (g) = Al_2_(MoO_4_)_3_ (s) ΔG = −2922.9 kJ/mol

### 3.4. Surface Morphology of the Oxidized Surface

**Figure 7:**
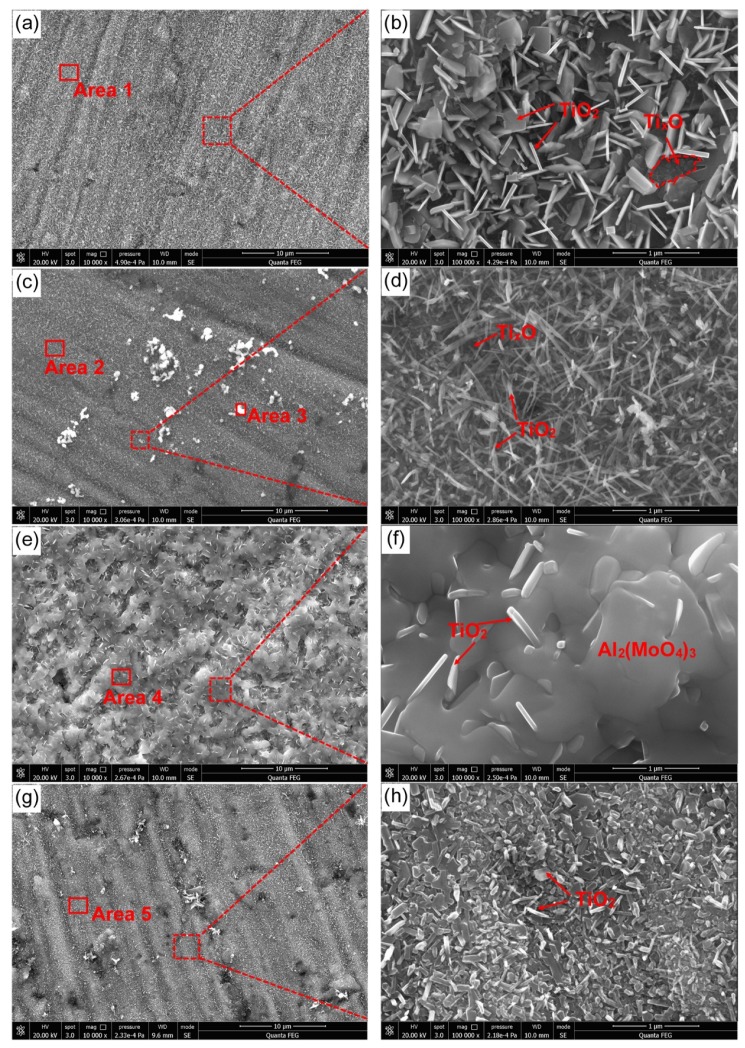
Surface morphology of the as-cast Ti-1100 based alloys for 650 °C oxidation during 100 h (**a**), (**b**) Ti-1100-0.5Nb; (**c**), (**d**) Ti-1100-1.0Nb; (**e**), (**f**) Ti-1100-1.5Nb; (**g**), (**h**) Ti-1100-2.0Nb.

Figure 7 displays the surface morphology of Ti-1100-xNb (x = 0.5, 1, 1.5, 2) alloys after oxidation for 100 h at 650 °C. The TiO_2_ with morphology of massive (Figure 7b) and acicular (Figure 7d) could be identified on the oxidation surfaces according to the EDS analysis results of Table 5. It is also found that the Ti-1100-0.5Nb alloy has finest acicular TiO_2_ and lower density porosity, which can inhabit the internal diffusion of O atoms and the growth of the oxide-film. With the increase of Nb content, some bright white particles with high oxide-layer density appeared on the surface, as shown in Figure 7c,d. The white oxide particles are the Mo-oxide which was proved by EDS in Table 5 (Area 3). The continuous oxide film can be found on the surface of Ti-1100-1.5Nb alloy. And it can be indexed as Al_2_(MoO_4_)_3_ according to the EDS (Table 5) as well as XRD (Figure 5) analyses. The Al_2_(MoO_4_)_3_ might be formed by the reaction between the MoO_3_ and the Al_2_O_3_, which was also confirmed by Heracleous et al. [26]. The atomic radius of Al, Ti, Mo is 0.143, 0.145 and 0.140 nm, respectively, which illustrate the Mo and Al solubilized in α-Ti lattice could cause the small distortion energy [27]. Table 5 (Areas 3 and 4) shows the enrichment of Mo and Al on the oxidized surface, so it is easy to form the MoO_3_ and Al_2_O_3_ on the near surface due to the lower ionization energy of Mo atoms and the higher covalent bond of Al-O [28,29]. Thus, a continuous Al_2_(MoO_4_)_3_ was formed on the matrix surface. It is found that the new nucleus is easy to form at the grain boundary, because it can promote more growth steps. The massive growth steps and rough growth interface can increase the nucleation rate and growth rate. Therefore, it can be concluded that the mass gains of Ti-1100-1.5Nb alloys exposed at 650 °C were mainly contributed by the Al_2_(MoO_4_)_3_ oxide-layer growth instead of the internal diffusion of O. Low valence oxides Ti_3_O, Ti_6_O have the same crystal structure (hcp) and similar lattice constant, and the O atoms easily dissolve in the octahedral of the α-Ti lattice to form an ordered structure [30]. Therefore, the Ti_3_O, Ti_6_O oxides can be identified by the α-Ti matrix (Figure 7b).

The XRD analysis presented in Figure 5 showed that NbO_2_ and Al_2_O_3_ oxides were also existed on the oxidation surfaces. However, they could not be recognized in Figure 7. The Nb dissolved in Ti-1100 alloy lattices can promote the precipitation of the silicide and the formation of TiO_2_ [7,31]. Therefore, the formation of NbO_2_ may be less and finer, which was invisible. In addition to the combination of Al_2_O_3_ and MoO_3_ to form Al_2_(MoO_4_)_3_ (Figure 7f), the Al_2_O_3_ oxide may also have a uniform fine particle distribution, which was not easy to find. As mentioned above, TiO_2_ has a different morphology. The crystal structure of rutil-TiO_2_ is the tetragonal with lattice constants of a = 0.4589 nm and c = 0.2954 [32,33]. Nb atoms may replace randomly Ti atoms at the corner of the tetragonal lattice, leading to the lattice expansion. Research pointed out that the energy barrier formed by Nb-O is lower than that of Ti-O [34]. Therefore, the crystal surface enriched by Nb was easy to capture the O atoms, which promotes the growth of this crystal planes. When Nb is randomly distributed in the TiO_2_ crystal, the crystal growth will show anisotropy, and the single crystal of TiO_2_ has massive structure. When Nb is distributed symmetrically on the crystal surface, crystal growth shows orientation, and the single crystal of TiO_2_ has a flake structure. TiO_2_ can grow with one dimensional along a certain direction to form acicular structure if no impurity atoms entered the lattice.

### 3.5. Cross-Section Morphologies and Elemental Profiles of the Oxide Layers

**Figure 8:**
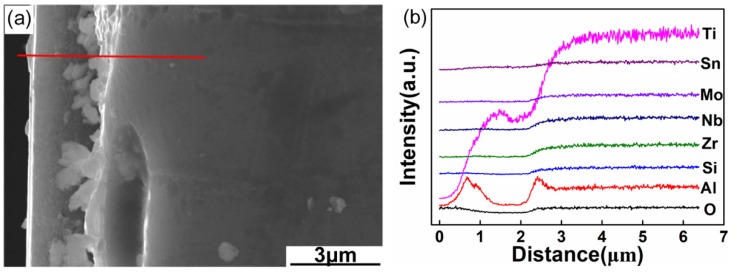

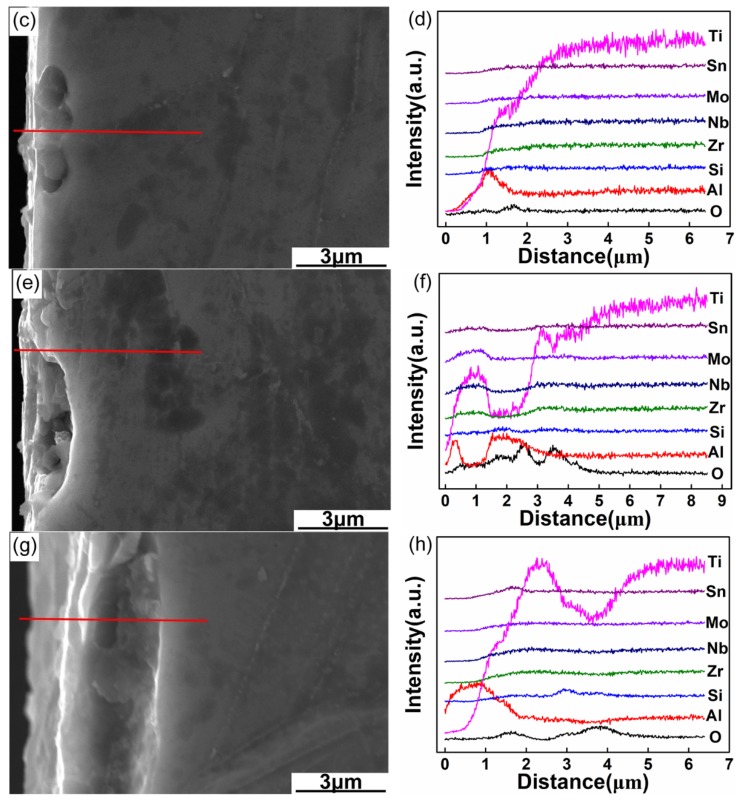
Cross-section morphologies and elemental distribution curves of the oxidation surfaces of the as-cast Ti-1100 based alloys after oxidation during 100 h at the temperature of 650 °C (**a**), (**b**) Ti-1100-0.5Nb; (**c**), (**d**) Ti-1100-1.0Nb; (**e**), (**f**), Ti-1100-1.5Nb; (**g**), (**h**) Ti-1100-2.0Nb.

Figure 8 shows the cross-section morphologies and elemental distribution curves of the oxidation surfaces of Ti-1100-xNb (x = 0.5, 1, 1.5, 2) alloys for 650 °C oxidation during 100 h. The oxide-layer is mainly composed of surface oxide-film and oxygen diffusion layer. Thickness of oxide-layer is 3.2, 1.8, 4.2 and 4.9 µm, respectively, with the increase of Nb content. It can be seen that the oxide layer increases firstly and then decreases, which was consistent with the weight gain law of the titanium alloys. Ti-1100-1.0Nb alloy has the minimum weight gains and oxide-layer thickness, which is conducive to the protection of slender TiO_2_ and the lower density porosity Ti_x_O (x = 3, 6) based microstructure (Figure 8d). Meanwhile, the enrichment Nb atoms can absorb the O atoms to refrain the internal diffusion of O atoms.

When the Nb content increases to 1.5 wt.%, there were the enriched layers of Mo, Nb and Al atoms near the oxide-surface, and its thickness reaches to 1.2 µm, as seen in Figure 8f. It can be identified as Al_2_(MoO_4_)_3_ layer according to the previous analysis. Some micropores in the Al_2_(MoO_4_)_3_ oxide-layer revealed its bad binding properties with the subsurface microstructure, which can lead to the peel off of the oxide-layer. It can also be found that Zr, Si have a slight fluctuation in Figure 8f,h. The enrichment Zr, Si and α-Ti in the interface may produce other compounds, such as (TiZr)_6_Si_3_ type silicide with the increase of Nb content [7], which caused the TiO_2_ oxide-film to crack and peel.

The growth rate of oxide-film can be determined by the Wagner model, as shown in Equation (7) [35]:

(7) $$J_{O^{2-}}\;=\;C_{O^{2-\;}}v_{O^{2-}}=\;-C_{O^{2-}}B_{O^{2-}}\left(\frac{\partial u_{O^{2-}}}{\partial x}+\;Z_{O^{2-}}F\frac{\partial \emptyset }{\partial x}\right)$$

where $J_{O^{2-}}$ is the diffusive flux of O ion, $C_{O^{2-}}$ is the concentrations of O ion, $v_{O^{2-}}$ is the diffusion rate of O ion, $B_{O^{2-}}$ is the mobility of O ion, $\frac{\partial u_{O^{2-}}}{\partial x}$ is the concentration gradient of O ion, $Z_{O^{2-}}$ is the particles charge, F is the Faraday constant, $\frac{\partial \emptyset }{\partial x}$ is electric potential.

The Wagner’s equation indicates that the driving force of internal diffusion of O atoms is electric potential gradient and concentration gradient. The diffusion rate of O ion is accelerated under the driving force of electric potential and concentration gradient. And there are a lot of oxygen vacancies in the segregation of elements or porosity area, which can promote the internal diffusion of O^2−^. On the contrary, the formation of compact oxide-film can reduce the O^2−^ concentration on the oxide surface. Nb can capture the O^2−^ in α-Ti lattice to form the ionic bond to reduce the electric potential gradient of O^2−^, which can inhibit the further diffusion of O atoms to the internal of matrix and effectively improve the oxidation resistance of titanium. Moreover, the interaction of Nb, O, Ti, and other elements can also retard the diffusion of O atoms into the alloys, which will further improve the oxidation resistance property.

In summary, the influence of adding Nb element on improving the high temperature oxidation resistance was quantitatively presented, with discussion on the role of Nb on surface oxide formation. However, the excessive addition of Nb may deteriorate the mechanical properties of the substrate, especially for the plasticity. So, how to balance the relationship between high temperature oxidation resistance and mechanical properties when choosing alloy composition should be considered in the future research.

## 4. Conclusions

1. With the increase of Nb content, both the α-Ti lattice parameters ‘a’ and ‘c’ of the as-cast alloys first rise and then decrease.
2. Adding Nb to Ti-1100 alloy did not change the microstructure classification of the alloys, and the space between α-laths decreases first and then increases with the increase of Nb content.
3. Nb can significantly improve the high temperature oxidation resistance. The oxidation behaviors of the alloys follow a parabolic kinetic model. The parabolic oxidation rate decreased firstly and then increased with the increase of Nb content. The Ti-1100-1.0Nb alloy has the lowest k_p_ value, which is 5.7 × 10^−13^.
4. Surface oxidation products are mainly composed of rutile-TiO_2_, Ti_x_O (x = 3, 6), NbO_2_ and Al_2_O_3_. The morphology of TiO_2_ is massive and acicular. Al_2_(MoO_4_)_3_ as the mainly oxide was first detected in the Ti-1100-1.5Nb alloys.
5. A novel oxidation resistance was obtained in the Ti-1100-1.0Nb alloy. The interaction of Nb, O, Ti, and other elements retarded the diffusion of O atoms into the alloys, which improved the oxidation resistance property as a result.

## Figures and Tables

**Table 1:** Chemical composition of as-cast Ti-1100-xNb alloys (wt.%).

	Al	Sn	Zr	Mo	Si	Nb	Ti
0.5 Nb	5.71	2.95	3.81	0.43	0.41	0.45	Balance
1.0 Nb	5.85	2.74	3.92	0.51	0.43	0.96	Balance
1.5 Nb	5.83	2.81	3.87	0.46	0.42	1.47	Balance
2.0 Nb	5.78	2.77	3.85	0.42	0.44	2.05	Balance

**Table 2:** Values of the oxidation reaction index n and the coefficient of determination R^2^ of Nb modified Ti-1100 alloys for 650 °C oxidation during 100 h.

Alloy	n	R^2^
Ti-1100-0.5Nb	1.955	0.996
Ti-1100-1.0Nb	1.792	0.989
Ti-1100-1.5Nb	1.919	0.995
Ti-1100-2.0Nb	2.000	0.995

**Table 3:** Values of the parabolic oxidation rate constant k_p_ and the coefficient of determination R^2^ of Nb modified Ti-1100 alloys for 650 °C oxidation during 100 h.

Alloy	k_p_ (g^2^cm^−4^s^−1^)	R^2^
Ti-1100-0.5Nb	9.222 × 10^−13^	0.990
Ti-1100-1.0Nb	5.749 × 10^−13^	0.981
Ti-1100-1.5Nb	7.496 × 10^−13^	0.988
Ti-1100-2.0Nb	8.733 × 10^−13^	0.989

**Table 4:** Lattice constant of α-Ti of the as-cast Ti-1100-xNb alloys before and after oxidized at 650 °C for 100 h.

	Composition	a	c	Δa	Δc
As-cast	0.5Nb	0.29289	0.46846	-	-
	1.0Nb	0.29357	0.46885	-	-
	1.5Nb	0.29346	0.46877	-	-
	2.0Nb	0.29333	0.46865	-	-
Oxidation condition	0.5Nb	0.29527	0.47737	0.00238	0.00891
	1.0Nb	0.29498	0.47342	0.00141	0.00457
	1.5Nb	0.29512	0.47441	0.00166	0.00564
	2.0Nb	0.29540	0.47475	0.00207	0.00610

**Table 5:** EDS analysis results of the oxidation surface marked in Figure 7 (wt.%).

Position	O	Al	Si	Nb	Mo	Ti	Zr	Sn
Area1	34.1	3.9	0.5	0.6	1.1	55.5	2.6	1.7
Area 2	34.6	3.2	0.0	0.0	0.0	59.9	0.7	1.6
Area 3	51.4	3.3	0.1	0.2	9.5	34.4	0.5	0.6
Area 4	44.1	6.9	0.2	0.7	19.2	27.2	0.9	0.8
Area 5	38.9	2.7	0.6	0.3	0.2	54.8	1.6	0.9
